# Fostering innovative behavior in health organizations: a PLS-SEM analysis of Norwegian hospital employees

**DOI:** 10.1186/s12913-021-06505-1

**Published:** 2021-05-18

**Authors:** Barbara Rebecca Mutonyi, Terje Slåtten, Gudbrand Lien

**Affiliations:** Inland School of Business and Social Sciences, Inland Norway University of Applied Sciences, Campus Lillehammer, Lillehammer, Norway

**Keywords:** Internal market-oriented culture, Psychological capital, Individual innovative behavior, Organizational commitment, Hospital employees

## Abstract

**Background:**

Health organization research is experiencing a strong refocus on employees’ individual innovative behavior (IIB), revealing that many of the influential factors at work remain uncertain. Hence, this study empirically examines fostering of hospital employees’ IIB by focusing on direct and indirect relationships of organizational culture (here labeled *internal market-oriented culture*, IMOC), psychological capital (PsyCap), and organizational commitment (OC).

**Methods:**

The study focused on a sample of 1008 hospital employees, using a partial least squares–structural equation modeling method to analyze and test the relationships hypothesized in this study. A multigroup comparison was performed to test the heterogeneity of personal characteristics. The indirect relationships of PsyCap were tested using mediator analyses.

**Results:**

Our results reveal that IMOC has a positive and significant correlation to employees’ PsyCap and IIB. PsyCap is directly related to IIB and indirectly related to IMOC and IIB. Furthermore, the study found that IIB is related to OC.

**Conclusions:**

This study extends the current debate on how IIB is fostered at work by examining PsyCap and IMOC as antecedents of IIB. The study has added to the IIB research area by examining the role of IIB on OC. The study is among the first attempts in its category to contribute to health organizations and managers by empirically examining the role of IMOC on employees’ PsyCap and IIB—and, in turn, their OC.

**Supplementary Information:**

The online version contains supplementary material available at 10.1186/s12913-021-06505-1.

## Background

Individual innovative behavior (IIB) has been termed a vital asset that enables organizations to thrive in a dynamic business environment [1]. Today, employees are progressively expected to “actively contribute to their organization’s success” [2], such as through idea generation and implementation [3]. Idea generation refers to creativity; in contrast, idea implementation refers to IIB and involves the successful implementation of creative ideas and solutions at work [4, 5]. Thus, IIB relies on both the generation of novel ideas (creativity) and their active application at work (innovation). IIB is understood to be the intentional use of a creative idea at work to perform tasks well, for the benefit of the group and the organization [6].

Worldwide, with the current technological advances and increased performance expectations for hospital employees [7], there has been an apparent increase in the challenges faced by health sector organizations [8]. Hospital employees’ IIB has been identified as a key factor in increasing innovation at work [7], improving effectiveness and performance [9] as well as efficiency [10]. Therefore, their creative and innovative solutions are crucial in responding to growing challenges [11].

As the study of IIB steadily gains attention [7, 12, 13], some consider that employees’ IIB is a key factor in improving overall job performance [14]. We build on this notion by investigating the direct and the indirect relationships of IIB among hospital employees. Specifically, this study examines how an organizational culture, here labeled *internal market-oriented cultur*e (IMOC) correlates to psychological capital (PsyCap) and IIB, and how, in turn, IIB correlates to organizational commitment (OC).

Despite the criticality of fostering IIB to promote innovation at work, particularly in health organizations [10, 14], few studies have explored its direct and indirect relationships among hospital employees [7, 14]. A literature review reveals three main areas that have been addressed repeatedly: job productivity, commitment, and empowerment. For instance, Xerri and Brunetto [15] examined the relationship of nursing employees’ commitment and organizational citizenship behavior on IIB. Other previous research explored the relationships of IIB on frontline hospitality employees’ feelings of joy [16]. Moreover, Knol and Van Linge [17] examined the correlation of structural and psychological empowerment on nursing employees’ IIB. To the best of the authors’ knowledge, this is a pioneering empirical study in health services research on the direct and indirect relationships of IIB among hospital employees. It responds to calls to investigate the conditions that encourage innovation and factors that relates to individual process innovation [7]. Few studies have adequately examined the IIB of hospital employees [7] and even fewer have empirically examined its direct and indirect relationships in a health sector context [8].

Consequently, more research on this topic is required. Specifically, recent research has argued that because IMOC is still in its infancy, more research on its correlation on employees’ PsyCap and IIB is necessary [18]. Moreover, a systematic review of innovation in health care by Länsisalmi et al. [19] has revealed the scarcity of studies of individual-level innovations. Nevertheless, it is clear that few empirical health service researchers have examined the links between IMOC, PsyCap, and IIB or the relationship between IIB and OC.

In response to calls for such research, this study has two unique implications. First, it expands the current theoretical knowledge pool and provides insight into the value of fostering IIB at work. Second, it provides further practical knowledge for managers desiring competitive advantage from their employees.

The present study makes three important contributions. First, it contributes new knowledge about fostering IIB in health organizations. Second, it empirically examines the close relationship between hospital employees’ perceptions of their organizations’ IMOC and their PsyCap and IIB. It offers new insights for health managers into the value of IMOC for engendering positive thoughts and actions. Third, the study contributes unique knowledge on the assumed causal relationship between IIB and OC. To the authors’ knowledge, no previous empirical health organization research has focused on these relationships. Consequently, this paper seeks to provide fresh knowledge on fostering hospital employees’ IIB at work.

Below, a conceptual model and relationships are proposed, followed by the theoretical background and hypotheses. Then, the methodology and results of the partial least squares–structural equation modeling (PLS-SEM) analyses are described. The paper concludes with a discussion of the empirical results and their implications for health managers, as well as the limitations of the study.

### Conceptual model

**Fig. 1:**
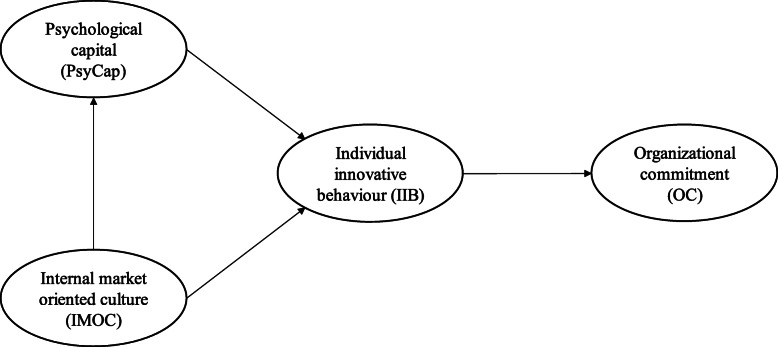
Conceptual Model of The Direct and Indirect Relationship of Hospital Employees’ IIB

As illustrated in Fig. 1, the conceptual model of this study includes both direct and indirect relationships. Specifically, this study proposes that IMOC is directly related to PsyCap and IIB, PsyCap is directly related to IIB, IIB is directly related to OC, and PsyCap is indirectly related to, or mediates the assumed relationship of IMOC and IIB. Therefore, Fig. 1 depicts the role of environmental factors such as IMOC on PsyCap, a personal resource. Figure 1 further illustrates how IMOC and PsyCap promote employees’ IIB. Furthermore, Fig. 1 shows how IIB promotes hospital employees’ OC. In addition, in the conceptual model of the study, we propose that a personal resource, PsyCap, mediates the assumed relationship between IMOC and IIB.

In the following sections, we discuss each of the elements and hypothesize linkages between them.

### Individual innovative behavior

The established and complex concept of IIB [4, 20–22] refers to the adoption, implementation, or use of novel ideas and solutions by employees to solve problems at work [20]. It is comprised of individual behaviors and intentions to generate, promote, and implement these ideas or solutions [6, 23]. Given the crucial role that it plays in overall organizational performance [24], success [25], competence [1], and effectiveness [26], fresh knowledge of hospital employees’ IIB is vital for modern health organizations to sustain their competitive advantage in the current turbulent environment [7].

Managers can improve their organizations’ competitive advantage in various ways [27], one of which is through employees’ IIB [28]. For instance, health organizations are advised to “encourage and develop the innovative potential of all their employees” [7]. In addition, because innovation is fundamental for an organization’s success and survival [8, 29], it is crucial to consider that in Industry 4.0 technologies, the psychological aspects of innovation such as IIB, are key strategic elements for successful global competition [30]. Therefore, organizations actively seek employees who are both innovative [7] and flexible in their approach to innovation [8]. Improving their psychological states and the internal culture of the organization are key factors in encouraging innovation, innovativeness, and IIB. This, in turn, brings fruitful results, such as greater commitment to the organization.

Although several individual factors shown in Fig. 1 have previously been linked to IIB in various studies [31–33], the direct and indirect relationships (such as PsyCap, IMOC and OC) of hospital employees’ IIB have yet to be studied. It is important to note that this limited research is detrimental because hospital employees are primary agents in implementing innovation at work [12, 34]. Furthermore, numerous studies have focused on nurses [35], doctors [14], or medical students [36]. However, studying IIB from a partial perspective limits our general understanding of its role on all hospital employees [7]. Carlucci et al. [7] expanded the focus of their study to include all hospital employees for the same reasons as this study. By including all employees, regardless of their role, one may capture not only the overall role of IIB but also the variance in each group (i.e., doctors and nurses). Health organizations pursue innovation through both management strategies [34] and their employees [12], which provides continuous growth and adaptation in the rapidly changing work environment [37]. Given the important roles of hospital employees in health organizations [14], specifically in terms of overall organizational innovation [7], it is vital to examine the direct and indirect relationships of IIB to understand how to engage them in active innovation processes.

Current empirical evidence shows that the dynamics between employees and organizations are far more complex than previously acknowledged [22], in that hospital employees do not always complete tasks in a straightforward fashion [14]. Consequently, Bos-Nehles et al. [20] and Mutonyi et al. [6] argued for further research on IIB at the individual level. Moreover, Slåtten et al. [30] recently called for an empirical exploration of the relationship of IMOC on employees’ IIB. Thus, there remains a significant gap in our current knowledge of IIB at work—specifically, the role of IMOC on employees’ PsyCap and IIB, the relationship of IIB on OC, and the assumed mediating role of PsyCap. The following sections will elaborate on the direct and indirect relationships of IIB, its relationships, as well as the hypotheses proposed in Fig. 1.

#### Psychological capital

Figure 1 indicates that PsyCap promotes IIB. Previous research contends that to improve overall work performance, employees should possess the personal attribute of PsyCap [38]. With its roots in positive psychology, PsyCap has previously been described as a meaningful and important construct in both psychological and organizational literature [38, 39]. As mentioned above, while prior research has mainly focused on three areas (productivity, commitment, and empowerment), it has largely been concerned with structural working conditions rather than personal conditions. Therefore, we devote our attention to personal characteristics such as PsyCap that may foster IIB at work. PsyCap is the relatively recently recognized concept of individuals’ positive assessment of their work settings and likelihood of success based on motivational efforts and perseverance [39]. PsyCap is understood to be “the positive psychological state of the individual towards positive development” [40], characterized by the acronym HERO, which stands for hope, efficacy, resilience, and optimism. Briefly, hope is a belief that determines an individual’s sense of purpose and success in a work role. Efficacy, or self-efficacy, is the conviction that one can mobilize motivation and cognitive resources to succeed at work tasks. Resilience is the ability to improvise and adapt in times of change. Finally, optimism is an individual’s permanence and pervasiveness. In other words, optimism is an individual’s positive expectations about the future, whereby one hopes for the best. In essence, PsyCap is who the individual is, either at work or personally. Thus, this study focuses on PsyCap to represent who health employees are at work. Luthans et al. [41] argue that PsyCap should encompass all of the HERO characteristics to capture employees’ positive psychological states fully. For instance, confidence, optimism, perseverance, and resilience are all positive states that can greatly relate to employees’ capability to innovate.

Although PsyCap has previously been studied in the health sector [42, 43], these studies focused strongly on factors such as well-being and burnout [44]. However, previous research has revealed PsyCap to be an important feature of employee attitudes, behavior, and performance [45], concluding that as a state attribute, it can relate to employees’ behaviors and attitudes towards implementing or promoting novel ideas. Other studies have explored PsyCap as a source of employees’ creativity [46], work engagement [47], and morale [48]. Nonetheless, the psychological state of employees can alter their feelings of psychological safety [40, 48] in promoting their ideas to others or seeking new working methods. It is important to point out that PsyCap in this study relates to a context and participants, namely hospital employees. Moreover, while examining the links between PsyCap, social capital, and the work performance of service sales representatives, Slåtten et al. [38] found positive direct and indirect correlation to innovative behavior. They call for further study in this area. Thus, this study improves the limited understanding of PsyCap among hospital employees [38]. In addition, examining working adults in the USA, Sweetman et al. [49] found that PsyCap and all its HERO components were positively related to creative performance. Sweetman et al. [49] recognized the infrequent attention given its importance at work, as it strongly relates to overall work performance. Moreover, studying business graduates, leaders, and employees, Lan [31] found PsyCap to be positively related to IIB. In this study, however, the focus on PsyCap is twofold: its role on hospital employees’ IIB and on employees in the Norwegian context. According to previous studies, it is plausible that there is a positive link between PsyCap and innovative behavior [32, 38, 49, 50]. Tcs the following hypothesis.

**Hypothesis 1:** *PsyCap is positively related to IIB*.

#### Internal market-oriented culture

A review by Scott et al. [51] revealed that organizational culture and structural change together produce improvements in quality and performance. An organizational culture is the shared values and beliefs that improve the understanding of operations [52]. For health organizations, these values and beliefs orient their employees toward achieving productivity, efficiency, development, and performance [31, 53, 54]. In the search for long-term profitability and successful implementation of an organizational culture, it has been proposed that IMOC should guide human resource management [55]. While market orientation entails an organizational culture where employees are committed to the continuous creation of value for their customers [56], internal market orientation describes one in which employees are internal customers, and the organization focuses on their wants and needs. Previous research has found that such organizations have generally benefited from improved overall performance [18, 31, 57]. Better job performance is related to long-term organizational success and competitive advantage [54]. We refer to the type of organizational culture that considers employees to be internal customers as an IMOC, which is comprised of three connected systems that create a logical flow of information [31]. The first, termed *internal market intelligence generation*, relates to gathering information on employees’ wants and needs. The second, termed *internal intelligence dissemination*, relates to whether managers understand these desires. The third, termed *response to internal intelligence* refers to the implementation of measures to satisfy them.

Previously, organizations have mainly focused on external factors (e.g., agents) that create and maintain superior value for their customers [55]. They use education programs and organizational changes to achieve the desired norm and learn from their efforts to develop and adapt. While both approaches increase the organization’s market orientation, it remains unclear how the internal market (e.g., employees) is related to their innovation capability. A recent study argued that health managers must pay attention to both approaches by considering both the external market (e.g., patients) and the internal market (e.g., employees) to build an organizational culture that drives innovation [31]. Research has proposed that organizational culture is a key variable in innovation success [58].

Therefore, this paper focuses on the importance of promoting IMOC in health organizations in relation to overall organizational performance and competitive advantage [18, 59]. Specifically, this study examines the value of IMOC on employees’ IIB at work, as there is a need to extend current knowledge of how it can be developed and promoted in health organizations.

In this study, IMOC is viewed in terms of hospital employees. Based on established literature in marketing [60–62] and organizational culture [62–64], IMOC is a rather new reconceptualization [31]. IMOC reflects the “more tangible or visible aspects of organizational culture … the observable norm-based behavior that constitutes organizational culture” [31]. Therefore, employees, especially hospital employees, must be “motivated not only by their own sense of self … but also by the contextual conditions of the organization” [24]. Previous research has examined the role of internal market orientation [65] and organizational culture [66] on IIB. As mentioned above, market orientation has traditionally focused on customers [65], whereas an internal market orientation focuses on employees’ wants and needs [67]. With IMOC, the attention is on employees’ perceptions of the degree of genuine care they receive from managers [31]. Previous research has explored the direct and indirect relationships of hospital frontline employees’ IMOC and found it to be positively related to the attractiveness of organizations to employees [31]. However, research into IMOC and its role on the work environment remains in its early stages [18, 31].

Because it is based on employees’ beliefs and expectations, IMOC can play a big role on their IIB in the work environment. For instance, previous research has argued that culture relates to and defines employee attitudes and behavior [1, 57, 63]. Moreover, organizational culture has been found to foster innovation and overall organizational performance [54]. For this reason, it is reasonable to assume that health employees’ perceptions of their organization’s IMOC is closely related to their willingness to implement new ideas and solutions because their needs and wants are addressed first. In addition, this study answers the call of Slåtten et al. [18, 30] to explore the role of IMOC in employees’ IIB. Conversely, IMOC is related to employee perceptions that an organization promotes the implementation of new ideas. Similar to the perceived relationship between the work environment and IIB [6], IMOC can be a powerful determinant of long-term organizational efficiency and performance.

As IIB refers to the adoption of novel ideas at work [20], the role of IMOC on employees’ perceptions of their organization being a desirable employer [31] is underestimated. To sustain organizational success and effectiveness in the long term [68, 69], it is essential to explore the potential correlations of IMOC on IIB. In other words, a good internal hospital culture that focuses on and cares about its employees can improve its efficiency and performance through its employees’ IIB. This relationship can be formally stated in the following hypothesis.

**Hypothesis 2:** *IMOC is positively related to IIB.*

As mentioned above, PsyCap (consisting of the HERO attributes) refers to the positive psychological state of individual development. PsyCap is the employees’ evaluation of who they are, their confidence, their dedication to their roles, their level of perseverance in the face of hardships, and their resilience [40]. Based on this evaluation, an employee may develop positive or negative associations, experiences, and attitudes towards their organization’s IMOC, with varying significance for their work life. To the best of the authors’ knowledge, this is one of the few novel studies in health service research to explore the direct role of IMOC on PsyCap, especially with a focus on hospital employees. Exploring this relationship is important because employees’ well-being is has a great impact on organizational performance and success [70]. In addition, IMOC has previously been found to add value to employees’ positive behaviors [65], showing that overall organizational culture is influential at all levels [71], especially the individual level [57]. For example, Luthans et al. [72] noted that a strong (internal) organizational culture can correlate internal behaviors positively or negatively. In addition, there has been a call to examine the role of IMOC on PsyCap in health organizations to add knowledge on and offer insight into its role and value [18]. Consequently, to build trust between the organizational leadership and individual employees, it is necessary to invest in and foster employees’ PsyCap proactively. Therefore, this study proposes the following hypothesis.

**Hypothesis 3:** *IMOC is positively related to PsyCap.*

#### Organizational commitment

In the literature, two approaches to the study of OC can be found: the one-dimensional approach and the multidimensional approach [73]. The one-dimensional approach focuses on the strength of the employees’ identification and involvement with the organization [73]. In contrast, in the multidimensional approach, OC is seen as a psychological state consisting of a combination of three factors: affective, continuance, and normative commitment [74, 75]. These three factors are often referred to as the three-component model of OC. A comparison of the popularity of the two approaches suggests that the multidimensional approach has been the most frequently used since it was introduced. OC in this study is rooted in the multidimensional approach. There are two main reasons for this choice. First, as mentioned above, this approach is most often used to study the OC of employees. Second, in the multidimensional approach, each of the three OC components is considered to be a psychological state. Studying OC as a trait implies that the construct is dynamic rather than static; therefore, it is changeable. This latter aspect is important as it is in line with one aim of this study, i.e., to explore the links between IIB and OC, and specifically whether IIB can have a positive correlation on the OC (trait) of employees. Although this study is rooted in the multidimensional approach to OC, it includes only one of the three components—affective commitment. The reason for this choice is that affective commitment in its nature and content is clearly the most positive of the three. This is true of OC whether from an employee or organizational point of view. Affective commitment refers to a psychological state that binds employees to the organization in a positive manner. Specifically, it is “the employee’s positive emotional attachment to the organization” [76]. Consequently, OC as an affective component captures a desire-based or “wants to” reason to commit to the organization. Studying OC as an affective component clearly contrasts with the other two components (in the multidimensional approach), which capture the obligation-based or “has to” (normative) or the “ought to” or cost-based (continuance) commitment [77]. Clearly, it is reasonable to assume that the affective component of OC is the most desirable type because it provides insight into employees’ perceptions of what is “good,” creating positive bonds with the organization. It is, therefore, not surprising that a substantial amount of research on OC has been concerned with its affective component [76].

As shown in Fig. 1, IIB is linked to OC. There are several examples in previous studies exploring the direct or indirect linkages between IIB and OC [33, 77, 78]. However, to the authors’ knowledge, no study has examined the linkage between IIB and OC in the domain of health services. Furthermore, previous studies on this topic have limited their focus to OC as a correlation to IIB. None has examined OC as a reversed correlation of IIB, as this study suggests. Although several plausible arguments have been proposed in the literature that OC relates to IIB, there are good reasons to expect the converse. This study defines OC as a “positive emotional attachment to the organization” [76]. Research has shown that emotions are always caused by something or someone [79]. Consequently, there must be one or more identifiable reason(s) for a person’s emotional attachment. Based on this logic, it is natural to expect triggering or motivational factors in the organizational sphere or context that are the true cause of OC. One such factor could be IIB.

IIB, as mentioned above, concerns employees’ freedom or autonomy to adopt or implement novel creative ideas to solve problems [25, 80]. Is it reasonable to assume that employees consider this freedom and autonomy to be positive and good? The converse would be a highly specific and controlled work situation in which employees had no freedom or autonomy to solve problems creatively. Naturally, employees’ IIB ranges from low to high. However, it may be assumed that the more the employees use their ability to experiment and be proactive in finding creative solutions, the more they perceive their organization to be an exciting and enjoyable workplace to which they will commit (in a positive way). Consequently, the expected relationship can be formally stated by the following hypothesis.

**Hypothesis 4:** *IIB is positively related to employees’ OC.*

#### The mediating role of psychological capital

PsyCap originated in the positive psychology literature [40], and mediation is prominent in psychological research [81]. In addition, it has previously been argued that to capture the actual internal mechanism to explain the linkage between IMOC and IIB [38], certain individual factors need to be included in the equation. For this reason, PsyCap is a proposed mediator for the assumed casual model Fig. 1. A mediating factor is in an intermediate position between an independent and a dependent variable. The conceptual model (Fig. 1) also proposes that PsyCap functions as a mediating factor in the relationship between IMOC and IIB. As argued by MacKinnon et al. [81], “attitudes cause intentions, which then cause behavior … memory processes mediate how information is transmitted into a response.” In other words, employees’ attitudes and beliefs, specifically about IMOC, will relate to how they perceive themselves at work, which in turn will affect their response—in this case to the implementation of novel ideas. This also implies that when organizational cultural values meet employees’ expectations [57], employees will feel more inclined to promote and implement novel solutions. Consequently, the following hypothesis is proposed.

**Hypothesis 5:** *The direct relationship between IMOC and IIB is mediated by PsyCap.*

## Methods

Data were collected in February 2020 from an online questionnaire survey of 2000 hospital employees in the inland counties of Norway. The health organization covers over 40 sites, with close to 10,000 employees, and is one of the largest health expert communities in its region. It services both psychiatric and somatic illnesses. Initial contact with the hospitals was sought through the Director of Research (DOR), followed by several meetings and email exchanges. With the help of the DOR, an information email was sent to division managers to inform their employees of the study. The survey information and URL were distributed by the DOR through emails to division managers, who passed them to their employees. To maintain participant anonymity and avoid nonresponse bias, the study used a platform called *Nettskjema*, which ensured full anonymity, such as automatic deletion of IP addresses when each participant had completed the survey. While there were some minor differences among divisions, it is important to note that the focus of the study is on individual behavior rather than divisional differences. As such, this study offers fresh insights on analyses at the individual level and the issues related to IIB among hospital employees. Through convenience sampling, the study collected a total of 1008 completed questionnaires—a response rate of 50.4%. Of the respondents in the study, 73% were women, reflecting the Norwegian context where the health sector is dominated by female workers and 84% of all employees [82] are women. In this study, about 37% of the hospital employees were under the age of 45, 77% worked full time, and over 55% had been employed at the organization for more than 10 years, amassing considerable work experience. The study’s respondents’ characteristics are summarized in Table 1.

**Table 1:** Personal characteristics of the study sample (*N* = 1008)

		Percent
Sex	Female	73.0
	Male	27.0
Work	Nurse	33.0
	Doctor	8.7
	Others (admin. Staff, other health professionals, etc.)	58.3
Employed	< 5 years	26.9
	6–10 years	18.0
	11–20 years	30.3
	> 20 years	24.8
Part-time or full-time	Part-time job	22.5
	Full-time job	77.5
Age	< 45 years	37.3
	46–55 years	32.2
	> 55 years	30.5

### Instruments

Four main instruments derived from the current literature were used to measure the conceptual model of the study (Fig. 1): PsyCap, IMOC, IIB, and OC. All participants responded to the validated survey items on a seven-point Likert scale (1 = strongly disagree to 7 = strongly agree). In addition to survey statements, the demographic characteristics shown in Table 1 were included. As the survey was conducted in the Norwegian language, several workshops with academic experts and employees were held to verify the back translation. Moreover, to ensure quality in the overall research design, two experts in the field, with 34 randomly selected hospital employees, completed a pre-test.

PsyCap was measured using four items adopted from Luthans et al. [39]. IMOC was measured using eight items from Slåtten et al. [30]. IIB was measured using five items from Janssen [83] and Scott and Bruce [4]. Finally, OC was measured using five items from Allen and Meyer [74]. All items used in this study, summarized in Table 2, were adjusted to the context of hospital employees in inland Norway. In addition, the survey used in this study is part of a larger survey research project focusing on various aspects of employee relations in health organizations. The statements used in this study are appended accordingly (see Additional file 1: Appendix 1).

**Table 2:** Latent variables and claims used in the study

Latent variable	Statement label	Statements
PsyCap	PsyCap1	I feel confident that I can set goals for myself in my work area.
	PsyCap2	I am optimistic about my future at this organization.
	PsyCap3	When faced with challenges in my job, I can find alternative solutions to them.
	PsyCap4	I can find alternative ways to achieve my goals.
IMOC	IMOC1	Employees have the opportunity to discuss their needs with management.
	IMOC2	Training is seen in the context of individual needs.
	IMOC3	Management spends time talking to their employees when needed.
	IMOC4	Management wants employees to enjoy their work.
	IMOC5	Management shows a sincere interest in any problems faced by employees.
	IMOC6	Management understands that personal problems may affect my performance.
	IMOC7	The division’s policies help meet employees’ individual needs.
	IMOC8	Management meets regularly to discuss issues related to employees’ challenges.
IIB	IIB1	I create new ideas to solve problems in my job.
	IIB2	I search out new working methods or techniques to complete my work.
	IIB3	I investigate and find ways to implement my ideas.
	IIB4	I promote my ideas so others might use them in their work.
	IIB5	I try out new ideas in my work.
OC	OC1	I am proud to tell others that I work here.
	OC2	I feel I belong in this organization.
	OC3	I feel personally attached to my organization.
	OC4	I envision a career at this organization.
	OC5	I want to continue my career here.

### Data analysis

The conceptual models and the hypothesized relationships were tested using PLS-SEM through SmartPLS 3 software [84]. The first step in evaluating the PLS-SEM results involved examining a set of criteria for the measurement model. Reflective measurement model specifications were applied, meaning that the direction of causality is from the constructs to their observed variables or claims. When the measurement model assessment was satisfactory, the next step was to assess the structural model. Then, mediating relationships were estimated and analyzed based on the PLS-SEM results. Finally, to check the robustness of the PLS-SEM results, we tested for observed and unobserved heterogeneity [85]. We followed the “rules of thumb” of Hair et al. [85, 86] to assess the quality of the measurement and structural model results.

## Results

### Measurement model

To assess the reflective measurement model, we examined convergent validity, internal consistency reliability, and discriminant validity. Convergent validity is the extent to which a variable correlates positively with alternative variables used to measure the same construct, and it was evaluated using variable loadings and average variance extracted (AVE). Internal consistency reliability provides estimates of a construct’s reliability based on the magnitudes of the intercorrelations of the observed variables, which were evaluated with composite reliability and Cronbach’s alpha. Discriminant validity is the extent to which a construct is distinct from other constructs and, as suggested by Hair et al. [86, 87], this was assessed with the heterotrait–monotrait (HTMT) ratio of correlations between constructs. The test is to ascertain that the 95% confidence interval of the HTMT value does not include the value of 1, as was the case for all four constructs in this study (IMOC, PsyCap, IIB, and OC). The remaining rule-of-thumb assessment criteria, all based on Hair et al. [86, 87], are reported in Table 3. As can be seen, all criteria were met, providing evidence of a measurement model that is both reliable and valid.

**Table 3:** Results of the measurement model for the constructs of PsyCap, IMOC, IIB, and OC

		Convergent validity		Internal consistency reliability		Discriminant validity
Latent variable	Claims label	Indicator reliability	AVE	Composite reliability	Cronbach’s alpha	HTMT criterion
Rule of thumb		Loading > 0.7	> 0.5	0.7–0.95	0.7–0.95	HTMT interval does not include 1
PsyCap	PsyCap1	0.82	0.74	0.92	0.88	Yes
	PsyCap2	0.82				
	PsyCap3	0.89				
	PsyCap4	0.90				
IMOC	IMOC1	0.84	0.73	0.95	0.94	Yes
	IMOC2	0.76				
	IMOC3	0.89				
	IMOC4	0.86				
	IMOC5	0.90				
	IMOC6	0.84				
	IMOC7	0.83				
	IMOC8	0.90				
IIB	IIB1	0.85	0.77	0.94	0.92	Yes
	IIB2	0.88				
	IIB3	0.90				
	IIB4	0.88				
	IIB5	0.87				
OC	OC1	0.85	0.72	0.93	0.90	Yes
	OC2	0.88				
	OC3	0.84				
	OC4	0.85				
	OC5	0.83				

Note: *AVE Average variance extracted, *HTMT* Heterotrait–monotrait ratio of correlations

### Structural model

**Fig. 2:**
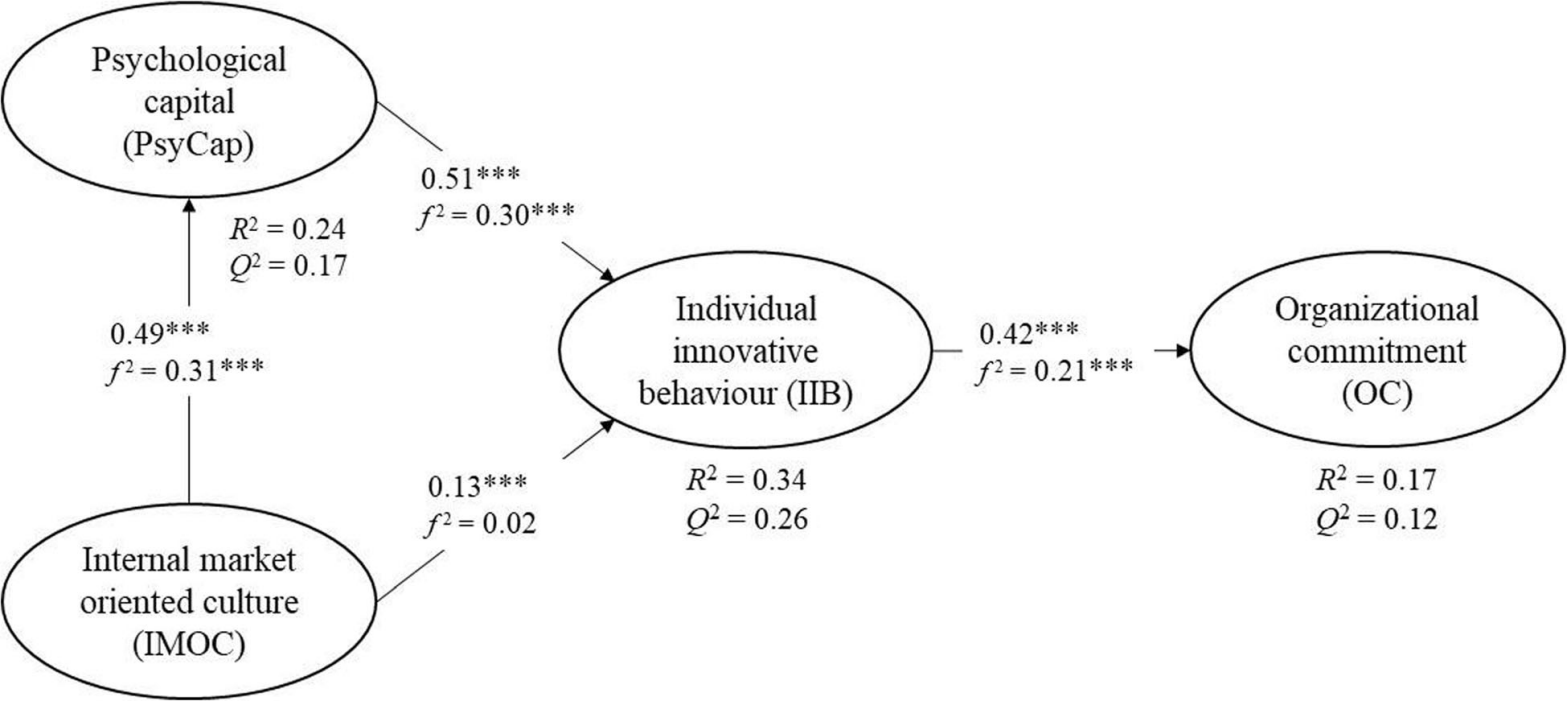
Results of the Structural Model of The Direct and Indirect Relationships of Hospital Employees’ IIB. Standardized coefficients (*** < 0.01)

Before the structural model was assessed, collinearity between the latent variables was examined using the variance inflation factor (VIF) values. All VIF values were lower than 2, indicating no multicollinearity problems. The direct relationships in the structural model are shown in Fig. 2. All direct relationships were statistically significant and positive. To identify any misspecifications in our PLS-SEM structural model, we followed three guidelines proposed by Hair et al. [86] for testing model fit indices against the empirical data. First, the model’s in-sample predictive power for the endogenous constructs was examined with the coefficient of determination, *R*^2^. Based on the rules of thumb of Hair et al. [86, 87], the *R*^2^ values for PsyCap (0.24) and IIB (0.34) were moderate and weak for OC (0.17).

Second, to evaluate changes in the *R*^2^ when a claim is omitted from its latent variable, effect size ƒ^2^ was used to determine the impact on latent variables. According to the guidelines of Hair et al. [86], the impact values differ. For example, a value of 0.02 is small, 0.15 is moderate, 0.35 is large, and values below 0.02 indicate no impact. The ƒ^2^ effect size values in our models were between 0.02 and 0.31, ranging from small to moderate (see Fig. 2).

Third, after assessing the model’s in-sample predictive power, we evaluated its out-of-sample predictive power Q^2^. As mentioned above, we used the PLS-SEM method to analyze our reflective model. Thus, to obtain Q^2^ values, the blindfolding method was used to obtain cross-validated redundancy values. Moreover, predictive relevance values differ when measuring Q^2^, where 0.02, 0.15, and 0.35 indicate small, moderate, or large effects, respectively. Our structural model showed moderate values for PsyCap (0.17), IIB (0.26), and OC (0.12), indicating overall medium predictive power (see Fig. 2).

The standardized path coefficient between PsyCap and IIB was the highest at 0.51, the second highest of 0.49 was between IMOC and PsyCap, and the third highest, 0.42, was between IIB and OC. There was also a statistically significant positive relationship between IMOC and IIB, but it was lower, at 0.13. The findings support all four proposed direct relationships (see Fig. 1) and the hypotheses of this study.

To test whether IMOC relates the proposed mediator variable PsyCap, resulting in a change in IIB in our PLS-SEM model, we used the mediation by bootstrapping method [86]. By testing the mediation relationships using PLS-SEM analysis, we can improve the understanding of the relationship between IMOC and IIB. Therefore, it makes sense to include individual positive assessments of the work setting, PsyCap, in modeling considerations. In testing the assumed mediator relationship of PsyCap, we followed the guidelines of Hair et al. [86] in regard to PLS-SEM models by bootstrapping the sampling distribution of indirect relationship. Based on variation accounted for (VAF) values, three types of mediation can be identified: complementary (partial), competitive (partial), and indirect-only (full mediation). VAF is the size of the indirect effect in relation to the total effect. Almost no mediation relationship is observed when VAF values are less than 0.20. VAF values > 0.20 and < 0.80 can be characterized as partial mediation and those ≥0.80 can be regarded as full mediation [86]. We tested the mediator relationship, i.e., whether PsyCap intervenes between IMOC and IIB, and found an indirect correlation of 0.25 (Table 4). We concluded that PsyCap partially mediated (complementary mediation) the relationship between IMOC and IIB.

**Table 4:** Test of mediation relationship of PsyCap

Effect^a^	Mediator^a^	Indirect effect^b^	Total effect^b^	VAF^c^	Mediator effect
IMOC → IIB	PsyCap	0.249^**^	0.383^***^	0.65	Partial

*IMOC* Internal market-oriented culture, *IIB* Individual innovative behavior, *PsyCap* Psychological capital

^a^Latent variables are IMOC, IIB and PsyCap

^b^** *p* < 0.05, *** *p* < 0.01 are significance levels

^c^*VAF* Variation accounted for

Additionally, while testing the hypothesized relationships, we examined the differences between identical models tested on different groups of respondents, with the objective of exploring any statistically significant differences. Therefore, we tested for observed heterogeneity [85] in three groups of respondents. First, we tested for observed heterogeneity [85] by dividing the sample into two groups: those employed at a hospital for ≤10 years and those employed for > 10 years. We then performed a multigroup analysis/permutation test. For these two groups, we found full measurement invariance and no statistically significant differences in the parameters of the structural model, suggesting that the data could be pooled. The test for unobserved heterogeneity using the finite mixture PLS-SEM technique [85] found the optimal number of segments to be one, suggesting that unobserved heterogeneity was not prevalent. Second, we completed a multigroup analysis to test for occupation differences. Accordingly, we tested for heterogeneity [85] using two subsamples: nurses and doctors. For these two groups, we found full measurement invariance. Both the permutation test and the multigroup analysis showed statistically significant differences in the parameters for the PsyCap–IIB relationship between these two groups. Third, we tested for differences between full-time and part-time employees. Both the permutation test and the multigroup analysis yielded statistically insignificant differences in the parameters of the structural model.

Ultimately, both the permutation test and the multigroup analysis for all three tests showed statistically insignificant differences in the parameters for the two subsamples, except for the relationship between PsyCap and IIB in the nurse and doctor groups (see Additional file 2: Appendix 2, Fig. A1). Nevertheless, the results of the overall model still applied, indicating robust results.

## Discussion

The results of our PLS-SEM analyses revealed five significant findings. First, IMOC was related to both PsyCap and IIB. Second, PsyCap was related to IIB. Third, IIB was related to OC. Fourth, PsyCap partially mediated the relationship between IMOC and IIB. Fifth, PLS-SEM was a strength of this study. The implications of these findings are discussed below.

### Theoretical implications

Our first finding that PsyCap is positively and significantly related to IIB highlights the importance of employees’ positive psychological development. Although this finding is consistent with previous studies of the role of PsyCap on IIB among graduate students [e.g., 32], these studies did not examine the role of PsyCap among hospital employees or its role in IIB. Additionally, our finding is consistent with those of Sun and Huang [88], who studied university teaching staff in China. Furthermore, the findings of Sameer [50] on Egyptian professionals was consistent with ours on the relationship between PsyCap and IIB. Consequently, our findings add new knowledge in the context of hospital employees in Norway. The findings of this study suggest that in organizations seeking long-term effectiveness and success, innovative employees’ IIB will be increased through PsyCap, rather than through prescribed work roles.

Second, our finding that IMOC has a positive and significant relationship with both PsyCap and IIB underscores the relevance of employees’ perceptions of their internal organizational culture. In particular, the visible and tangible characteristics of an organizational culture require not only training and opportunities but also a genuine interest in employees’ work life to satisfy their individual needs and wants. Because IMOC does this, they are motivated by the organizational environment. Although prior research [31] found IMOC to be related to the attractiveness of organizations to employees, it did not examine its role in their PsyCap or IIB. This study is the first to examine the role of IMOC for all types of health organization employees, complementing previous findings regarding the central role of IMOC in employees’ perceptions that their organization promotes innovation.

Third, our finding that IIB is positively and significantly related to OC indicates that employees’ positive emotional attachment to their organization is a result of their cognitive motivation to implement novel ideas at work. This study, which is among the first to examine the role of IIB on OC, underscores the key role of hospital employees’ IIB on their desire to remain in the organization. Additionally, our focus on IIB as a variable related to OC sheds light on the driving motivational force of IIB on employees’ “positive emotional attachment to the organization” [76], while providing fresh and valuable insights into the role of IIB in OC at work.

Fourth, our findings showed that PsyCap partially mediates and strengthens the relationship between IMOC and IIB. The findings are consistent with the assumed causal model shown in Fig. 1, but can also be consistent with a number of other causal models. In other words, the contextual conditions, or IMOC, have a positive and significant correlation to the psychological state of hospital employees, or PsyCap, which in turn positively correlates to IIB. Previous research has examined PsyCap as a mediating factor between management support and readiness for change [89] and between organizational innovation climate and IIB [90]. However, our focus on the relationship between IMOC and IIB underscores the importance of focusing on and caring about employees to foster IIB.

Fifth, the use of PLS-SEM method to examine the hypothesized relationships illustrated in Fig. 1 is a strength of this study because it furthers our understanding of the current complexity of the interactions between health organizations and their employees that affect innovation at work. PLS-SEM analysis makes a valuable contribution concerning the fostering of IIB in health organizations. Health managers are situated in complex environments where they must often attend to a range of issues, so they regularly depend on their subordinates to deliver high quality patient care [91]. In addition, several health organizations are situated in environments that may be hostile to innovative behavior because of time constraints and the scrutiny of risk-taking behavior related to patient care [14]. Therefore, it is important to gain a better understanding of the complex interactions in fostering IIB at work, as this study does, using the PLS-SEM method and multigroup analysis [85]. The strength of this approach is that it is possible to conduct a permutation test while avoiding errors such as distributional assumptions [92]. Previous studies have called for methodical research using PLS-SEM [93], with a specific call for more mediation [94] and multigroup analyses [92]. Although the results of the multigroup analysis (see Additional file 2: Appendix 2) demonstrated that the overall model still applied, indicating robust results, there were noteworthy differences between nurses and doctors in terms of the PsyCap–IIB relationship.

### Practical limitations

The empirical findings of this study depicted in Fig. 2 and the mediation analysis shown in Table 4 suggest that health organizations must seek to understand the direct and indirect relationships of employees’ IIB, in addition to understanding the role of IIB on OC. While we acknowledge the quandary that this poses for health organizations in terms of resource management and quality service [8], the increased attention to the importance of IIB at work suggests that health managers should encourage individual implementation of novel ideas to promote autonomy in the delivery of high-quality health care.

Furthermore, the findings of this study suggest that IIB may be fostered through the psychological state of employees and an internally coherent IMOC equipped to develop the framework and the competence necessary to motivate IIB at work and sustain competitive advantage. In contrast to an emotion, PsyCap is a state-like resource that is flexible and open to development [40, 95]. As such, managers can invest in it to improve their organization’s effectiveness and performance. This study shows the strength of PsyCap, both in its direct relationships to IIB and how it relates indirectly to IMOC and IIB. This is particularly important given the various calls for health organization research to help health managers understand the implications of IIB [15, 17]. These implications include how to foster IIB at work [7], strategically invest in employees’ psychological state [96], promote a culture where employees perceive management to be present [31], and develop strategic bonds to increase their emotional attachment to their organization [97]. The increasing need for innovative employees [6], especially in health organizations [7], has resulted in managers seeking strategic sustainable solutions to current challenges [9, 10]. Consequently, these findings provide fresh insights into the creation of organizational settings that instill the HERO attributes to promote idea implementation at work. In addition, health managers are advised to nurture employees by listening, showing interest, and discussing issues with them. As important as it is for employees to feel emotionally motivated to implement novel ideas, it is also vital that health managers develop positive perceptions of their organizations to strengthen employee commitment.

This study also found that the indirect relationship of PsyCap partially mediated the assumed relationship between IMOC and IIB. The implications for health managers are that IMOC predicts positive PsyCap, which in turn raises IIB among hospital employees. Failing to recognize the predictive power of this personal resource can reduce the HERO attributes. Thus, this study reveals the importance of possessing tools and skills to develop ideal workplace environments, or IMOCs, that improve employees’ practical IIB and boost their cognitive PsyCap, which in turn generates commitment to the organization. Consequently, health managers should be suitably trained to ensure the desired outcomes for the organization.

The findings reported in this study expand our current understanding of the intricacy of fostering IIB in health organizations. This knowledge is particularly relevant to policymakers [98] who view innovation as indispensable for organizational adaptation, survival, and long-term success [99]. Based on these findings, policymakers are advised to conduct internal surveys and use these results to create guidelines and regulations to promote an enabling environment. This, in turn, will provide health managers with the appropriate tools and methods to drive innovation at work, while increasing employees’ confidence, optimism, resilience, and efficacy. Such methods will improve employees’ capability to resolve problems and provide them with an organizational culture they may be proud of and to which they will feel a sense of belonging.

### Limitations and future research

The limitations of the current study offer opportunities for future research. First, although we followed the steps and guidelines for a cross-sectional study [100], the design has various limitations. For example, the data obtained in this study were collected at one time from one region: inland Norwegian counties. Therefore, the results have limited generalizability to other health organizations. Scholars who undertake future cross-sectional studies are advised not only to test the causality of the relationships in this study but also to collect data from a range of sites. However, support for the partial mediation of PsyCap in the relationship between IMOC and IIB suggests that our results are not entirely attributable to method bias. Nevertheless, to minimize method bias, future research may broaden the sample across regions and nations.

Second, the online survey in this research may suffer from self-selection bias, in addition to the possibility of reversed causality. In addition, IIB in this study was measured using self-report measures, a limitation that has previously been criticized because of the possibility of shared response bias among the variables [101, 102]. However, recent studies suggest that surveys can be used in research exploring direct and indirect relationships in an assumed causal model [103]. In addition, several past studies have elicited employees’ perceptions of their IIB [12, 25, 104–106] through self-report measures. Nevertheless, while this study maintained respondent anonymity to minimize self-report bias [103, 107], future research can gather data at different times with varying foci. For example, future research can explore self-reported data on employees’ IIB, but also examine whether actual innovation has taken place to compare actual and perceived innovative behavior.

Third, although IMOC and PsyCap are grounded in previous research, we did not measure their discrepancies and variations to explain the positive relationship with IIB. As the first study of its kind to examine the relationship between hospital employees’ IIB and OC, we consider the findings to be stepping stones to further exploration. In addition, while previous research has focused on IIB as an outcome variable [21], there is limited understanding of its workplace outcomes, especially in health organizations. Given the strategic role of employees’ IIB in the overall innovation success of an organization [108], it will be crucial for researchers to uncover competitive advantage.

Fourth, the findings in this study receive further credibility not only from our focus on the positive aspects of employee behavior, such as IIB, but also because we included all types of hospital employees to investigate the role of PsyCap. Therefore, future research can explore further its mediating role and discrepancies in this context, as well as the role of health managers in the implementation of IIB.

## Conclusions

In this study, we proposed and tested a conceptual model to analyze the direct and indirect relationships of IIB among hospital employees in inland Norwegian counties. Our findings revealed that IMOC correlates to both PsyCap and IIB. Furthermore, PsyCap directly relates to employees’ IIB. We examined the relationship of IIB and OC at work, and found a positive and significant relationship. In addition, we explored the indirect role of PsyCap and found that it partially mediates the assumed relationship between IMOC and IIB. We hope the findings of this study inspire future research into how health managers can invest in employees’ PsyCap, develop an IMOC with long-term benefits, foster IIB at work, and improve employees’ emotional attachment to their organization. In this way, health managers will be equipped with the required skills and competence to develop capabilities to sustain competitive advantage.

## Supplementary Information

**Additional file 1: Appendix 1.** Questionnaire Developed For This Study.

**Additional file 2: Appendix 2.** Multigroup Analysis. **Figure A1.** Multigroup analysis of: 1) number of years employed at the hospital (upper panel); 2) part-time or full-time (in the middle); and 3) occupation type (lower panel) (** < 0.05, *** < 0.01).

## Data Availability

The datasets used and/or analyzed during the current study are available from the corresponding author on reasonable request.

## Abbreviations

AVE: Average variance extracted
DOR: Director of research
*f*^2^: Effect size
HTMT: Heterotrait–monotrait
IIB: Individual innovative behavior
IMOC: Internal market-oriented culture
OC: Organizational commitment
PLS-SEM: Partial least squares–structural equation modeling
PsyCap: Psychological capital
Q^2^: Predictive relevance
VAF: Variation accounted for
VIF: Variance inflation factor

## References

[1] Korzilius H, Bücker JJLE, Beerlage S (2017). Multiculturalism and innovative work behavior: the mediating role of cultural intelligence. Int J Intercult Relat.

[2] Carnevale JB, Huang L, Crede M, Harms P, Uhl-Bien M (2017). Leading to stimulate employees’ ideas: a quantitative review of leader–member exchange, employee voice, creativity, and innovative behavior. Int Assoc Appl Psychol.

[3] Anderson N, Potočnik K, Zhou J (2014). Innovation and creativity in organizations: a state-of-the-science review, prospective commentary, and guiding framework. Aust J Manag.

[4] Scott SG, Bruce RA (1994). Determinants of innovative behavior: a path model of individual innovation in the workplace. Acad Manag J.

[5] Amabile TM (1988). A model of creativity and innovation in organizations. Res Organ Behav.

[6] Mutonyi BR, Slåtten T, Lien G (2020). Organizational climate and creative performance in the public sector. Eur Bus Rev.

[7] Carlucci D, Mura M, Schiuma G (2020). Fostering employees’ innovative work behavior in healthcare organisations. Int J Innov Manag.

[8] Glover W, Nissinboim N, Naveh E (2020). Examining innovation in hospital units: a complex adaptive systems approach. BMC Health Serv Res.

[9] Asurakkody TA, Shin SY (2018). Innovative behavior in nursing context: a concept analysis. Asian Nurs Res.

[10] Saleem M, Tufail MW, Atta A, Asghar S (2015). Innovative workplace behavior, motivation level, and perceived stress among healthcare employees. Pak J Commer Soc Sci.

[11] Chang S-C, Lee M-S (2008). The linkage between knowledge accumulation capability and organizational innovation. J Knowl Manag.

[12] Mutonyi BR, Slåtten T, Lien G (2020). Empowering leadership, work group cohesiveness, individual learning orientation and individual innovative behaviour in the public sector: empirical evidence from Norway. Int J Public Leadersh.

[13] Bos-Nehles A, Bondarouk T, Nijenhuis K (2017). Innovative work behaviour in knowledge-intensive public sector organizations: the case of supervisors in the Netherlands fire services. Int J Hum Resour Manag.

[14] Oppi C, Bagheri A, Vagnoni E (2019). Antecedents of innovative work behaviour in healthcare: does efficacy play a role?. Int J Public Sect Manage.

[15] Xerri MJ, Brunetto Y (2013). Fostering innovative behaviour: the importance of employee commitment and organisational citizenship behaviour. Int J Hum Resour Manag.

[16] Slåtten T (2011). Antecedents and effects of employees’ feelings of joy on employees’ innovative behaviour. Int J Qual Serv Sci.

[17] Knol J, Van Linge R (2009). Innovative behaviour: the effect of structural and psychological empowerment on nurses. J Adv Nurs.

[18] Slåtten T, Lien G, Lupina E, Gravingen KA (2019). Promoting an internal market-oriented culture (IMOC) in healthcare services. J Serv Sci Res.

[19] Länsisalmi H, Kivimäki M, Aalto P, Ruoranen R (2006). Innovation in healthcare: a systematic review of recent research. Nurs Sci Q.

[20] Bos-Nehles A, Renkema M, Janssen M (2017). HRM and innovative work behaviour: a systematic literature review. Pers Rev.

[21] Burns DJ (2007). Toward an explanatory model of innovative behavior. J Bus Psychol.

[22] Janssen O (2005). The joint impact of perceived influence and supervisor supportiveness on employee innovative behaviour. J Occup Organ Psychol.

[23] Suseno Y, Standing C, Gengatharen D, Nguyen D (2019). Innovative work behaviour in the public sector: the roles of task characteristics, social support, and proactivity. Aus J Pub Admin.

[24] Yuan F, Woodman RW (2010). Innovative behavior in the workplace: the role of performance and image outcome expectations. Acad Manag J.

[25] Battistelli A, Montani F, Odoardi C, Vandenberghe C, Picci P (2014). Employees’ concerns about change and commitment to change among Italian organizations: the moderating role of innovative work behavior. Int J Hum Resour Manag.

[26] Pieterse AN, Van Knippenberg D, Schippers M, Stam D (2010). Transformational and transactional leadership and innovative behavior: the moderating role of psychological empowerment. J Organ Behav.

[27] Li X, Zheng Y (2014). The influential factors of employees’ innovative behavior and the management advices. J Serv Sci Manag.

[28] Lee A, Legood A, Hughes D, Tian AW, Newman A, Knight C (2020). Leadership, creativity and innovation: a meta-analytic review. Eur J Work Organ Psychol.

[29] Duradoni M, Di Fabio A (2019). Intrapreneurial self-capital and sustainable innovative behavior within organizations. Sustainability..

[30] Slåtten T, Lien G, Svenkerud PJ (2019). The role of organizational attractiveness in an internal market-oriented culture (IMOC): a study of hospital frontline employees. BMC Health Serv Res.

[31] Lan X (2019). How psychological capital promotes innovative behavior: a mutilevel modeling. Am J Ind Bus Manag.

[32] Marques T, Galende J, Cruz P, Ferreira MP (2014). Surviving downsizing and innovative behaviors: a matter of organizational commitment. Int J Manpow.

[33] Slåtten T, Mutonyi BR, Lien G (2020). The impact of individual creativity, psychological capital, and leadership autonomy support on hospital employees’ innovative behaviour. BMC Health Serv Res.

[34] Kim S-J, Park M (2015). Leadership, knowledge sharing, and creativity: the key factors in nurses’ innovative behaviors. J Nurs Admin.

[35] Yan D, Wen F, Li X, Zhang Y (2020). The relationship between psychological capital and innovation behavior in Chinese nurses. J Nurs Manag.

[36] Çinar F, Toker K (2019). An examination of the effect of loneliness on the innovative behavior of health science faculty students. Chin Med J.

[37] Amabile TM, Conti R, Coon H, Lazenby J, Herron M (1996). Assessing the work environment for creativity. Acad Manag J.

[38] Slåtten T, Lien G, Horn CMF, Pedersen E (2019). The links between psychological capital, social capital, and work-related performance—a study of service sales representatives. Total Qual Manage Bus.

[39] Luthans F, Youssef CM, Avolio BJ (2007). Psychological capital: developing the human competitive edge.

[40] Luthans F, Youssef-Morgan CM (2017). Psychological capital: an evidence-based positive approach. Annu Rev Organ Psych Organ Behav.

[41] Luthans F, Youssef-Morgan CM, Avolio BJ (2015). Psychological capital and beyond.

[42] Laschinger HKS, Fida R (2014). New nurses burnout and workplace wellbeing: the influence of authentic leadership and psychological capital. Burn Res.

[43] Luthans KW, Jensen SM (2005). The linkage between psychological capital and commitment to organizational mission: a study of nurses. J Nurs Admin.

[44] Luthans F, Youssef CM, Sweetman DS, Harms PD (2013). Meeting the leadership challenge of employee well-being through relationship PsyCap and health PsyCap. J Leadersh Org Stud.

[45] Avey JB, Reichard RJ, Luthans F, Mhatre KH (2011). Meta-analysis of the impact of positive psychological capital on employee attitudes, behaviors, and performance. Hum Resour Dev Q.

[46] Rego A, Sousa F, Marques C, Cunha MPE (2012). Authentic leadership promoting employees’ psychological capital and creativity. J Bus Res.

[47] du Plessis M, Boshoff AB (2018). Authentic leadership, followership, and psychological capital as antecedents of work engagement. J Psychol Afr.

[48] Paek S, Schuckert M, Kim TT, Lee G (2015). Why is hospitality employees’ psychological capital important? The effects of psychological capital on work engagement and employee morale. Int J Hosp Manag.

[49] Sweetman D, Luthans F, Avey JB, Luthans BC (2011). Relationship between positive psychological capital and creative performance. Can J Adm Sci.

[50] Sameer YM (2018). Innovative behavior and psychological capital: does positivity make any difference?. J Econ Manage.

[51] Scott T, Mannion R, Davies H, Marshall M (2003). The quantitative measurement of organizational culture in health care: a review of the available instruments. Health Serv Res.

[52] Cameron KS, Quinn RE (2011). Diagnosing and changing organizational culture: based on the competing values framework.

[53] Cerqueira ADS, Mainardes EW, De Oliveira JLB (2018). Dimensions of internal market orientation related to job satisfaction and appreciation in Brazilian healthcare service. J Health Manag.

[54] Naranjo-Valencia JC, Jiménez-Jiménez D, Sanz-Valle R (2016). Studying the links between organizational culture, innovation, and performance in Spanish companies. Rev Latinoam Psicol.

[55] Lings IN, Greenley GE (2005). Measuring internal market orientation. J Serv Res.

[56] Narver JC, Slater SF, Tietje B (1998). Creating a market orientation. J Mark-Foc Manage.

[57] Mesfin D, Woldie M, Adamu A, Bekele F (2020). Perceived organizational culture and its relationship with job satisfaction in primary hospitals of Jimma zone and Jimma town administration, correlational study. BMC Health Serv Res.

[58] Büschgens T, Bausch A, Balkin DB (2013). Organizational culture and innovation: a meta-analytic review. J Prod Innov Manag.

[59] Baneshi E, Rezaei B (2013). Depicting favorite organizational culture: an empirical case study. Manage Sci Lett.

[60] Gounaris SP (2006). Internal-market orientation and its measurement. J Bus Res.

[61] Yu Q, Asaad Y, Yen DA, Gupta S (2016). IMO and internal branding outcomes: an employee perspective in UK HE. Stud High Educ.

[62] Ouchi WG, Wilkins AL (1985). Organizational culture. Annu Rev Sociol.

[63] Hofstede G (1998). Attitudes, values and organizational culture: disentangling the concepts. Organ Stud.

[64] Alvesson M (2013). Understanding organizational culture.

[65] Chao C-Y, Lin Y-S, Cheng Y-L, Liao S-C (2011). A research on the relationship among market orientation, absorptive capability, organizational innovation climate and innovative behavior in Taiwan’s manufacturing industry. Afr J Bus Manag.

[66] Sinha S, Priyadarshi P, Kumar P (2016). Organizational culture, innovative behaviour and work-related attitude: role of psychological empowerment. J Work Learn.

[67] Lings IN (2004). Internal market orientation: construct and consequences. J Bus Res.

[68] Hansen JA, Pihl-Thingvad S (2019). Managing employee innovative behaviour through transformational and transactional leadership styles. Public Manag Rev.

[69] Hartnell CA, Ou AY, Kinicki A (2011). Organizational culture and organizational effectiveness: a meta-analytic investigation of the competing values framework’s theoretical suppositions. J Appl Psychol.

[70] Harun S, Sürücü L, Maşlakçı A (2019). On the relation between leadership and positive psychological capital in the hospitality industry. Int J Bus.

[71] Li W, Bhutto TA, Nasiri AR, Shaikh HA, Samo FA (2018). Organizational innovation: the role of leadership and organizational culture. Int J Public Leadersh.

[72] Luthans F, Vogelgesang GR, Lester PB (2006). Developing the psychological capital of resiliency. Hum Resour Dev Rev.

[73] Porter LW, Steers RM, Mowday RT, Boulian PV (1974). Organizational commitment, job satisfaction, and turnover among psychiatric technicians. J Appl Psychol.

[74] Allen NJ, Meyer JP (1990). Affective, continuance, and normative commitment to the organization: an examination of construct validity. J Occup Psychol.

[75] Wasti SA (2005). Commitment profiles: combinations of organizational commitment forms and job outcomes. J Vocat Behav.

[76] Jafri MH (2010). Organizational commitment and employee’s innovative behavior: a study in retail sector. J Manag Res.

[77] Meyer JP, Allen NJ (2004). TCM employee commitment survey academic users guide 2004.

[78] Slåtten T, Mehmetoglu M (2011). What are the drivers for innovative behavior in frontline jobs? A study of the hospitality industry in Norway. J Hum Resour Hospit Tourism.

[79] Slåtten T (2011). Emotions in service encounters from the perspectives of employees and customers.

[80] Bos-Nehles AC, Veenendaal AA (2017). Perceptions of HR practices and innovative work behavior: the moderating effect of an innovative climate. Int J Hum Resour Manag.

[81] MacKinnon DP, Fairchild AJ, Fritz MS (2007). Mediation analysis. Annu Rev Psychol.

[82] Jensen RS, Øistad BS (2019). Det kjønnsdelte arbeidsmarkedet på virksomhetsnivå [The gender-segregated labour market at the workplace level].

[83] Janssen O (2000). Job demands, perceptions of effort–reward fairness and innovative work behaviour. J Occup Organ Psychol.

[84] Ringle CM, Wende S, Becker J-M (2015). SmartPLS 3.

[85] Hair JF, Sarstedt M, Ringle CM, Gudergan SP (2018). Advanced issues in partial least squares structural equation modeling.

[86] Hair JF, Hult GTM, Ringle C, Sarstedt M (2017). A primer on partial least squares structural equation modeling (PLS-SEM).

[87] Hair JF, Risher JJ, Sarstedt M, Ringle CM (2019). When to use and how to report the results of PLS-SEM. Eur Bus Rev.

[88] Sun Y, Huang J (2019). Psychological capital and innovative behavior: mediating effect of psychological safety. Soc Behav Pers.

[89] Kirrane M, Lennon M, O’Connor C, Fu N (2017). Linking perceived management support with employees’ readiness for change: the mediating role of psychological capital. J Chang Manag.

[90] Hsu ML, Chen FH (2015). The cross-level mediating effect of psychological capital on the organizational innovation climate–employee innovative behavior relationship. J Creat Behav.

[91] Hannemann-Weber H, Kessel M, Budych K, Schultz C (2011). Shared communication processes within healthcare teams for rare diseases and their influence on healthcare professionals’ innovative behavior and patient satisfaction. Implement Sci.

[92] Sarstedt M, Henseler J, Ringle Christian M, Marko S, Manfred S, Charles RT (2011). Multigroup analysis in partial least squares (PLS) path modeling: alternative methods and empirical results. Measurement and research methods in international marketing. Measurement and research methods in international marketing. Advances in International Marketing.

[93] Ringle CM, Sarstedt M, Mitchell R, Gudergan SP (2020). Partial least squares structural equation modeling in HRM research. Int J Hum Resour Manag.

[94] Sarstedt M, Hair JF, Nitzl C, Ringle CM, Howard MC (2020). Beyond a tandem analysis of SEM and PROCESS: use of PLS-SEM for mediation analyses!. Int J Mark Res.

[95] Peterson SJ, Luthans F, Avolio BJ, Walumbwa FO, Zhang Z (2011). Psychological capital and employee performance: a latent growth modeling approach. Pers Psychol.

[96] Bitmiş MG, Ergeneli A (2015). How psychological capital influences burnout: the mediating role of job insecurity. Procedia Soc Behav Sci.

[97] Vandenberghe C, Bentein K, Panaccio A (2017). Affective commitment to organizations and supervisors and turnover: a role theory perspective. Aust J Manag.

[98] Tricco AC, Zarin W, Rios P, Nincic V, Khan PA, Ghassemi M (2018). Engaging policy-makers, health system managers, and policy analysts in the knowledge synthesis process: a scoping review. Implement Sci.

[99] Wang Z, Wang N (2012). Knowledge sharing, innovation and firm performance. Expert Syst Appl.

[100] Levin KA (2006). Study design III: cross-sectional studies. Evid Based Dent.

[101] Jackson M, Cox DR (2013). The principles of experimental design and their application in sociology. Annu Rev Sociol.

[102] Thye SR, Webster M, Sell J (2014). Logical and philosophical foundations of experimental research in the social sciences. Laboratory experiments in the social sciences.

[103] Thrane C (2020). Surveyeksperimentet: et underutnyttet forskningsdesign for sosiologisk kausalanalyse. Nor Sosiol Tidsskr.

[104] Carmeli A, Meitar R, Weisberg J (2006). Self-leadership skills and innovative behavior at work. Int J Manpow.

[105] Nazir S, Shafi A, Atif MM, Qun W, Abdullah SM (2019). How organization justice and perceived organizational support facilitate employees’ innovative behavior at work. Empl Relat.

[106] Vinarski-Peretz H, Carmeli A (2011). Linking care felt to engagement in innovative behaviors in the workplace: the mediating role of psychological conditions. Psychol Aesthet Creat Arts.

[107] Schmitt N (1994). Method bias: the importance of theory and measurement. J Organ Behav.

[108] De Jong J, Den Hartog D (2010). Measuring innovative work behaviour. Creat Innov Manag.

