# Supplementary material for: Fostering innovative behavior in health organizations: a PLS-SEM analysis of Norwegian hospital employees
Source: BMC Health Serv Res. 2021 May 18;21:470. doi: 10.1186/s12913-021-06505-1 (PMC8130526; doi:10.1186/s12913-021-06505-1)
Supplement: Supplementary file 1 — Additional file 1: Appendix 1. Questionnaire Developed For This Study. [file 12913_2021_6505_MOESM1_ESM.docx]

**APPENDIX 1: QUESTIONNAIRE DEVELOPED FOR THIS STUDY**

Survey text information:

You are invited to participate in this survey on factors affecting employee-relationship in health organizations. The purpose is to study various conditions that affect your daily job. Your participation in this survey will help managers and your organization gain a better understanding of matters that are important to your job.

Only individuals 18 years and over can participate in the survey.

The survey contains statements that you are free to choose point scales that are best for you. The study must be conducted in one setting and cannot be saved for later attempts.

Respond to the claims focusing on your immediate work environment, for example; your department or work unit.

It will take you approx. 8 - 10 minutes to complete the survey.

Be aware that the survey can be answered on PC, tablet and mobile (use landscape mode).

Voluntary participation

Participation in the survey is voluntary and anonymous. If you respond, you have consented to participate. If you change your mind in the middle of your response, you do not need to submit the form. After the form is submitted, the responses cannot be retracted because they are submitted anonymously and cannot be traced back to you, nor through indirect information or hidden electronic traces such as IP address.

The Norwegian Center for Research Data AS (NSD) and the Data Protection Officer (DPO) has considered that the processing of personal data in this project complies with the privacy regulations.

If you have questions or need more information, please contact the project manager: Barbara Rebecca Mutonyi

I give my consent to participate in the survey by proceeding, and I confirm that I have read and understood the information above

Personal characteristics:

I am:

A leader

A subordinate

Sex:

Male

Female

Age:

Please fill in the year you are born (e.g. 1985)

How long have you been with the current organization:

Less than a year

1 – 5 year(s)

6 – 10 years

11 – 20 years

21 – 30 years

31 – 40 years

40+ years

Employment type:

Part-time

Full-time

Other

Please state your staff role:

Nurse

Doctor

Administrative staff

Professional development staff

Other health staff

Other

Highest level of education:

High school

Bachelor Degree

Master Degree

Doctor of Philosophy degree

Other

Total work experience in public health organizations:

Less than a year

1 – 5 year(s)

6 – 10 years

11 – 20 years

21 – 30 years

31 – 40 years

40+ years

Please confirm the health organization you belong to:

Please state your division:

Please state you administrative area (if applicable):

Claims/statements:

Statements with a 7-point scale:

1= Strongly disagree

2 = Disagree

3 = Somewhat disagree

4 = Neither agree or disagree

5 = Somewhat agree

6 = Agree

7 = Strongly agree

Internal market-oriented culture (IMOC)

Management refers to your immediate management / manager. For example, department manager. On a 7-point scale from 1 (Strongly disagree) to 7 (Strongly agree), please rate how you agree or disagree with the following statements:

Employees have the opportunity to discuss their needs with management.

Training is seen in the context of individual needs.

Management spends time talking to their employees when needed.

Management wants employees to enjoy their work.

Management shows a sincere interest in any problems faced by employees.

Management understands that personal problems may affect my performance.

The division’s policies help meet employees’ individual needs.

Management meets regularly to discuss issues related to employees’ challenges.

Psychological capital (PsyCap)

On a 7-point scale from 1 (Strongly disagree) to 7 (Strongly agree), please rate how you agree or disagree with the following statements:

I feel confident that I can set goals for myself in my work area.

I am optimistic when it comes to my future at this organization.

When faced with challenges in my job, I can find alternative solutions to them.

I can find alternative ways to achieve my goals.

Individual innovative behavior (IIB)

On a 7-point scale from 1 (Strongly disagree) to 7 (Strongly agree), please rate how you agree or disagree with the following statements in your work:

I create new ideas to solve problems in my job.

I search out new working methods or techniques to complete my work.

I investigate and find ways to implement my ideas.

I promote my ideas so others might use them in their work.

I try out new ideas in my work.

Organizational commitment (OC)

The organization refers to the organization you are currently affiliated with. On a 7-point scale from 1 (Strongly disagree) to 7 (Strongly agree), please rate how you agree or disagree with the following statements:

I am proud to tell others that I work here.

I feel I belong in this organization.

I feel personally attached to my organization.

I envision a career at this organization.

I want to continue my career here.
