# Supplementary material for: Fostering innovative behavior in health organizations: a PLS-SEM analysis of Norwegian hospital employees
Source: BMC Health Serv Res. 2021 May 18;21:470. doi: 10.1186/s12913-021-06505-1 (PMC8130526; doi:10.1186/s12913-021-06505-1)
Supplement: Supplementary file 2 — Additional file 2: Appendix 2. Multigroup Analysis. Figure A1. Multigroup analysis of: 1) number of years employed at the hospital (upper panel); 2) part-time or full-time (in the middle); and 3) occupation type (lower panel) (** < 0.05, *** < 0.01). [file 12913_2021_6505_MOESM2_ESM.docx]

**APPENDIX 2: MULTIGROUP ANALYSIS**


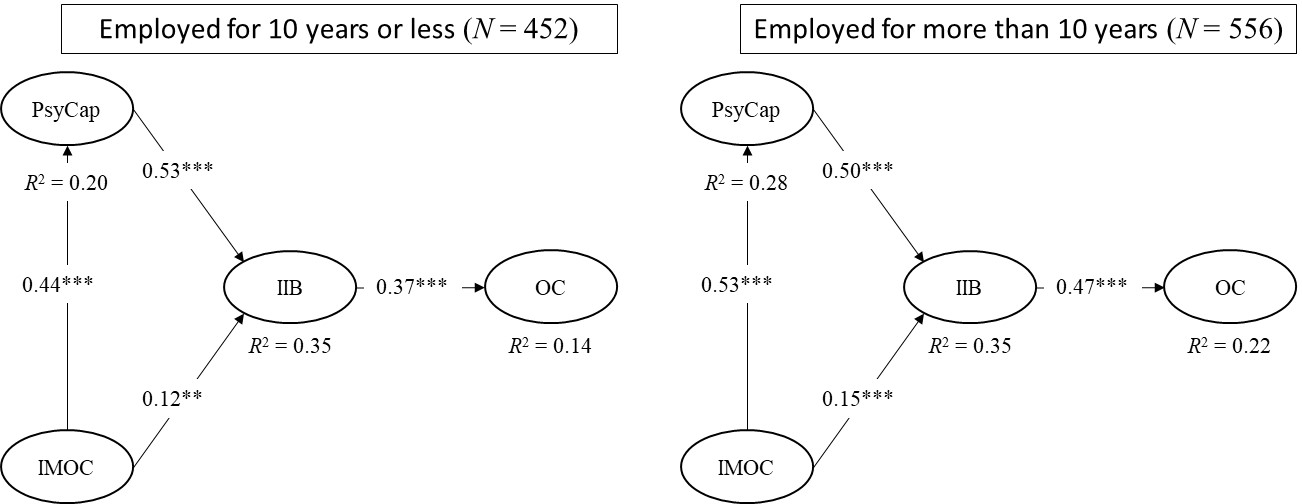


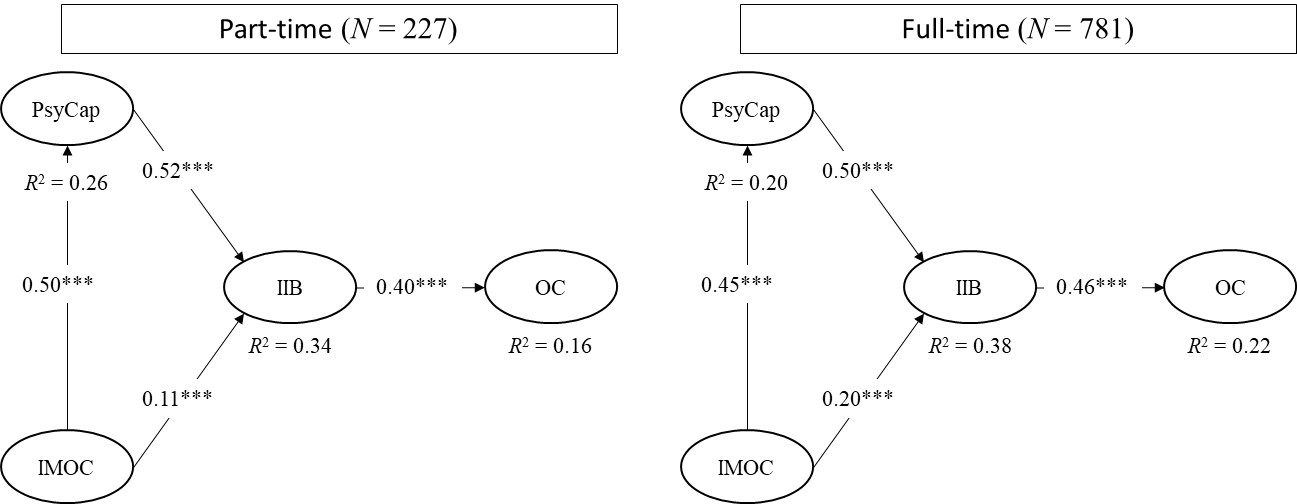


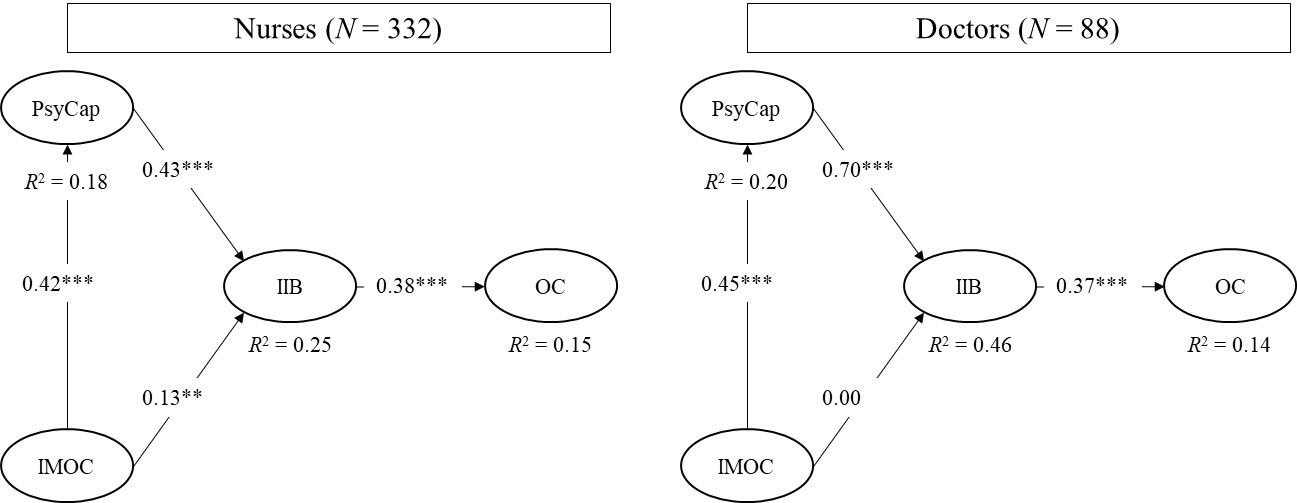


**Fig. A1.** Multigroup analysis of : 1) number of years employed at the hospital (upper panel); 2) part-time or full-time (in the middle); and 3) occupation type (lower panel) (** <0.05, *** <0.01)
